# Echocardiographic predictors of outcome in severe aortic stenosis patients with preserved or reduced ejection fraction

**DOI:** 10.1007/s00392-023-02350-w

**Published:** 2024-01-22

**Authors:** Victoria Sokalski, Dan Liu, Kai Hu, Stefan Frantz, Peter Nordbeck

**Affiliations:** 1 Department of Internal Medicine I, University Hospital Würzburg, Oberdürrbacher Str. 6, 97080 Würzburg, Germany; 2 Comprehensive Heart Failure Center, University Hospital Würzburg, Oberdürrbacher Str. 6, 97080 Würzburg, Germany

**Keywords:** TAVI, Heart failure with reduced ejection fraction, Heart failure with preserved ejection fraction, Echocardiography, Survival

## Abstract

**Aims:**

Transcatheter aortic valve implantation (TAVI) has emerged as the treatment of choice for many patients with severe symptomatic aortic stenosis. We sought to identify the echocardiographic predictors of 30-day and 1-year outcomes after TAVI in patients with preserved or reduced left ventricular ejection fraction (LVEF).

**Methods:**

This single-centre study included 618 aortic stenosis patients (mean age 82 ± 6 years, 47.1% male; 74.8% LVEF > 50%) who underwent balloon-expandable TAVI between July 2009 and October 2018 in our hospital. All patients completed at least 6 months of follow-up by medical history review or telephone interview (median 24, quartiles 12–42 months). The primary endpoint was all-cause death.

**Results:**

All-cause mortality rate was 5.2% (LVEF > 50%: 4.3% vs. LVEF ≤ 50%: 7.7%, *p* = 0.141) at 30 days and 15.4% (LVEF > 50%: 14.7% vs. LVEF ≤ 50%: 17.3%, *p* = 0.443) at 12 months post TAVI. Overall all-cause mortality rate was 45.1% (LVEF > 50%: 44.6% vs. LVEF ≤ 50%: 46.8%, *p* = 0.643). Mean survival time post TAVI was 51 months [95% CI (48; 55)]. In TAVI patients with LVEF > 50%, multivariate Cox regression analysis revealed several independent predictors for increased risk of death after adjusting for echocardiographic and clinical covariates: TAPSE (≤ 17 vs. > 17 mm, HR 1.528, *p* = 0.016) and sPAP (> 30 vs. ≤ 30 mmHg, HR 1.900, *p* = 0.002) for overall mortality, E/E′ septal for 30-day mortality (> 21 vs. ≤ 21, HR 14.462, *p* = 0.010) and 12-month mortality (> 21 vs. ≤ 21, HR 1.881, *p* = 0.026). In TAVI patients with LVEF ≤ 50%, no independent echocardiographic predictors for outcome could be identified.

**Conclusions:**

LVEF is not a predictor of short- and long-term mortality after TAVI. In patients with preserved LVEF, left ventricular filling pressure (E/E´), systolic pulmonary artery pressure (sPAP), and TAPSE are echocardiographic risk factors for increased mortality post TAVI.

**Graphical abstract:**

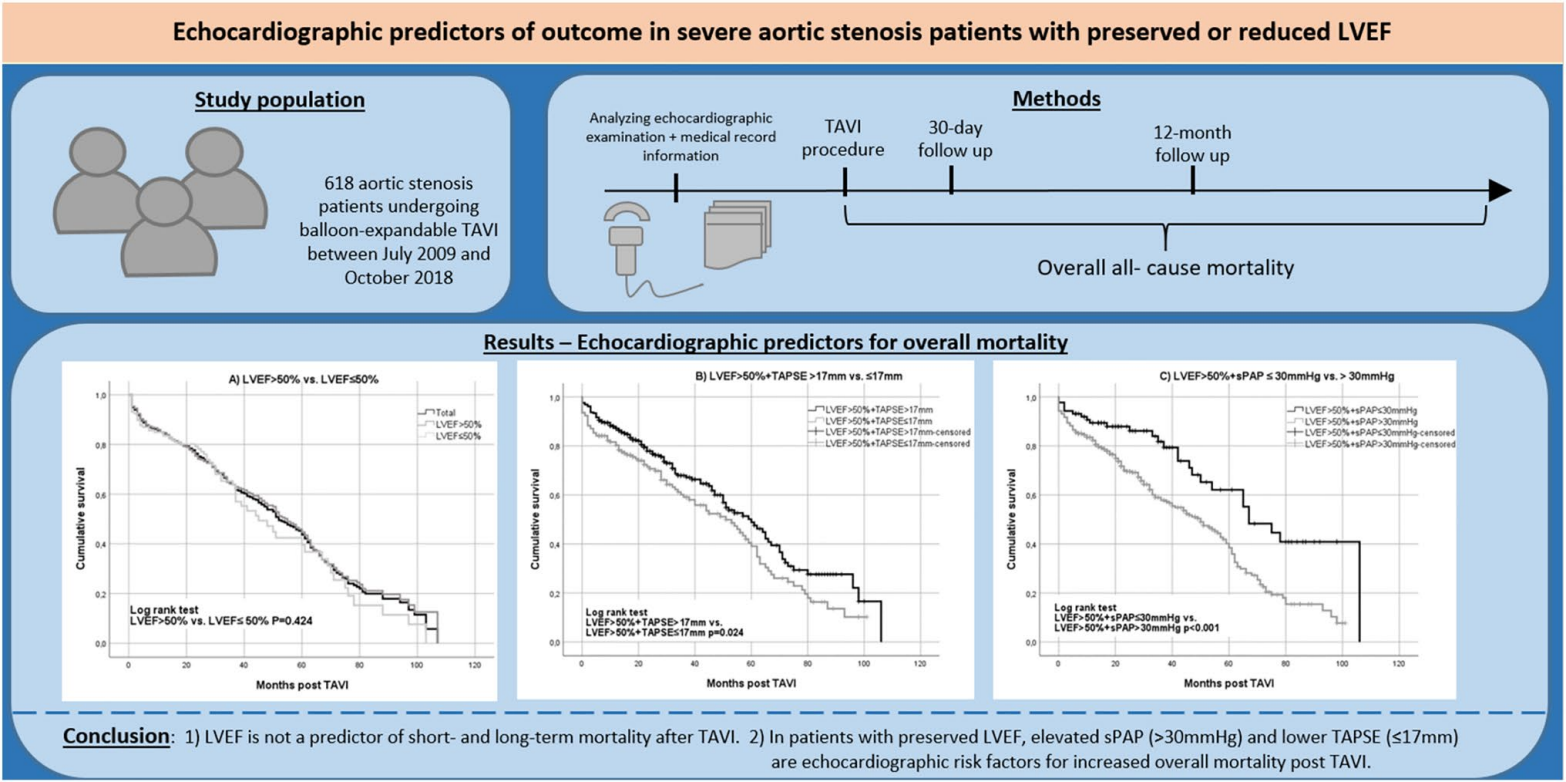

**Supplementary Information:**

The online version contains supplementary material available at 10.1007/s00392-023-02350-w.

## Introduction

Aortic stenosis (AS) is the most common valvular disease in Europe requiring intervention—open-heart surgery or transcatheter aortic valve implantation (TAVI) [1]. As the ageing population increases and demographics change, we see a growing prevalence of severe degenerative AS; a meta-analysis and modelling study estimated a prevalence of 3.4% in patients ≥ 75 years, meaning approximately 4.9 million patients in European countries and 2.7 million in North America [2, 3]. Nevertheless, valvular diseases still represent an underestimated public health issue even though research and development have led to ongoing improvements in patient care and treatment safety [4]. Since its first performance in 2002, TAVI has emerged as the treatment of choice in a growing proportion of patients with severe AS, mainly because it is less invasive and therefore has reduced procedural risks. After a consecutive evaluation by the heart team, as recommended in the ESC/EACTS Guidelines for the Management of Valvular Heart Disease [1], the patient is either suited to TAVI or conventional surgical aortic valve replacement (SAVR). As displayed in the German Aortic valve RegistrY (GARY,) the number of TAVI procedures has been increasing exponentially in recent years, exceeding SAVR since 2013 [5]. Nevertheless the TAVI procedure, as any other intervention, entails specific risks, e.g. paravalvular regurgitation, bleeding, stroke, myocardial infarction, acute kidney injury or death.

Simultaneously, the number of patients with heart failure is also increasing, with approximately half of the patients presenting with a reduced left ventricular ejection fraction (LVEF ≤ 50%) and the other half presenting with a preserved left ventricular ejection fraction (LVEF > 50%) [6]. An increasing number of patients present with severe aortic stenosis and concomitant heart failure. As shown in a large meta-analysis, patients with a preserved LVEF have a generally lower risk of death regardless of age, sex and aetiology of HF [7]. The extent to which the LVEF affects patient-specific complications and overall outcome in patients with severe aortic stenosis and whether recommendations for therapy should be stratified according to LVEF remain unclear. Furthermore, we sought to identify relevant clinical and echocardiographic predictors of short-term (30-day) and long-term (1-year) outcomes in patients with preserved or reduced left ventricular ejection fraction (LVEF) after TAVI.

## Methods

### Study population

**Fig. 1:**
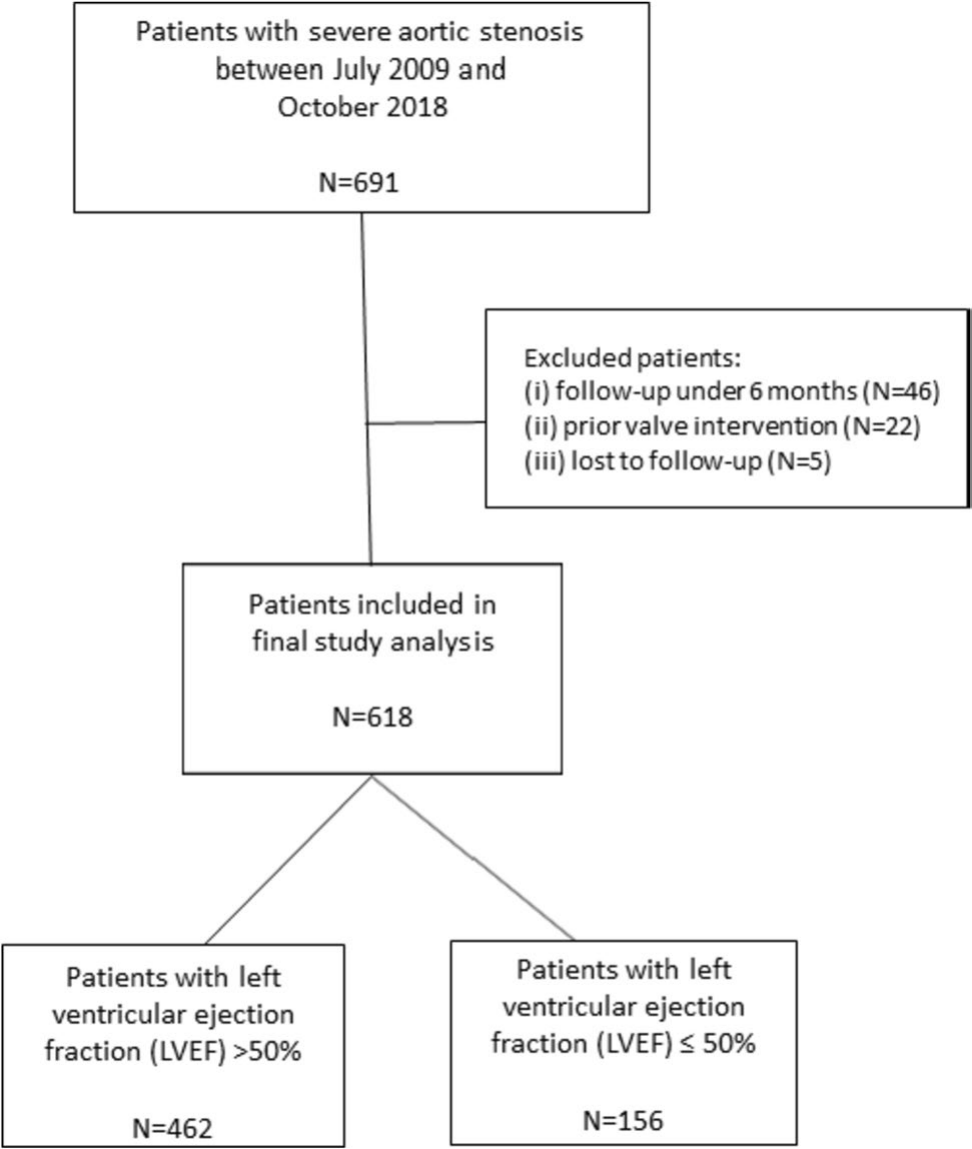
Study population

This single-centre study included 618 patients who underwent balloon-expandable TAVI between July 2009 and October 2018 in our hospital. Initially, we screened 691 patients and excluded 73 based on the following criteria: (i) follow-up less than 6 months (n = 46), (ii) prior valve intervention (n = 22), and (iii) loss to follow-up (n = 5). A total of 74.8% (462/618) of patients presented with a left ventricular ejection fraction (LVEF) > 50%, and 25.2% (156/618) presented with a LVEF ≤ 50% (Fig. 1). Severe aortic stenosis was diagnosed according to the recommendations from the European Association of Cardiovascular Imaging and the American Society of Echocardiography [8]. Our heart team (including interventional cardiologists, heart-thoracic surgeons, radiologists and anaesthetists) ultimately decided whether the patient was suitable for open-heart surgery or TAVI.

The investigation was conducted in accordance with the principles outlined in the Declaration of Helsinki and approved by the Local Ethics Committee at the University of Würzburg. Written informed consent was obtained from all patients or their guardians prior to study start.

### Standard echocardiographic measurements

Echocardiographic measurements were made offline regarding the last examination prior to TAVI using ECHO-Pac (GE Vingmed Ultrasound AS, Horton, Norway). All measurements were performed according to the guidelines of the European Association of Cardiovascular Imaging and the American Society of Echocardiography [8]. In summary, our comprehensive assessments included left ventricular (LV) end-diastolic dimension (LVEDD) and end-diastolic thickness of the septum (IVSd) and posterior wall (LVPWd) measured in M-mode in the parasternal long-axis view. The diameter, area and volume of the right atrium (RAD, RAA, RAV) were measured in the right ventricular (RV)-focused apical four-chamber view, while the area and volume of the left atrium (LAA and LAV) were examined in apical four-chamber and two-chamber views. LVEF was calculated using the biplane Simpson method in both apical four-chamber and two-chamber views. For further assessment of systolic function, mitral annular plane systolic excursion septal and lateral (MAPSE) as well as tricuspid annular plane systolic excursion (TAPSE) were measured in the apical four-chamber view in M-mode. Diastolic function was assessed using pulsed wave Doppler for the measurement of mitral inflow velocities E (early) and A (atrial) in the apical four-chamber view. Consequently, the deceleration time (DT) and the E/A ratio were used to evaluate LV diastolic dysfunction according to the filling pattern. Using tissue Doppler, early diastolic mitral annular velocity (e′) septal and lateral of the mitral annulus was measured, followed by calculation of septal, lateral, and average E/e′. Finally, systolic pulmonary artery pressure (sPAP) can be calculated with the help of the Bernoulli equation consisting of estimated central venous pressure (CVP) and the peak tricuspid regurgitation jet velocity (TRVmax) measured by continuous wave Doppler.

### Clinical data and outcome

To analyse clinical data, we evaluated patients’ medical record information focusing on cardiovascular risk factors (e.g., obesity, peripheral vascular disease, atrial fibrillation, dyslipidaemia). In addition, we checked laboratory data and medication prescriptions. All patients completed at least 6 months of follow-up by medical history review or telephone interview. In five cases we could not get any information about survival (time) or death dates so that these five patients are defined as loss to follow-up. Mortality data for all other patients were ascertained independently from clinical follow-up via clinical visit, telephone call with patients, their relatives or general practitioners or document from residential authorities. The primary endpoint was defined as all-cause death.

### Statistical analysis

The present data were analysed using SPSS Statistics Version 27 (IBM, Somers, New York, USA). Continuous variables are expressed as the mean ± standard deviation or median with interquartile range. Data were first checked for normality distribution by the Shapiro‒Wilk test. Continuous variables with normal distributions were compared using unpaired Student’s t tests, and data with skewed distributions were tested by nonparametric Mann–Whitney U tests. Categorical variables are expressed as counts and percentages; differences between groups were compared using Pearson’s chi-square test.

To identify predictors of all-cause death, we used univariate and multivariate Cox proportional hazard regression models. Hazard ratios (HRs) with 95% confidence intervals (CIs) were calculated and reported. Univariate Cox proportional hazard analysis was performed to investigate potential confounders associated with post-interventional mortality. Subsequently, variables with *p* values < 0.05 in univariate were used to establish multivariate Cox regression by the stepwise backward elimination process based on the likelihood-ratio. In the final model, all significant clinical and echocardiographic parameters (*p* < 0.05) were included to determine independent predictors. Spearman correlation was used to detect the potential collinearity between variables. When r > 0.6, collinearity was considered to exist and consequently the variables were not included into the Cox regression model. Predictors remaining significant were then transformed into binary variables using ROC curves (Supplement Fig. S3) and Youden’s index to make identification of high-risk patients easier and more concrete for clinical practice.

Survival curves were estimated by the Kaplan‒Meier method and compared by using the log-rank test. A two-tailed probability value of less than 0.05 was considered significant.

## Results

### Baseline clinical and echocardiographic characteristics

Patients were divided into two groups: preserved left ventricular ejection fraction (LVEF > 50%, n = 462) and reduced left ventricular ejection fraction (LVEF ≤ 50%, n = 156).

The mean age of the patients with a preserved LVEF was 82 ± 5 years and 81 ± 6 years in patients with a reduced LVEF. The latter group consisted of significantly more men (62.8% vs. 41.8%, *p* < 0.001) and showed a higher mean EuroSCORE II (9.3% ± 6.1% vs. 5.9% ± 4.6%, *p* < 0.001) whereas the distribution of patients with a New York Heart Association (NYHA) functional class III-IV was similar between both groups (69.0% in the LVEF > 50% group vs. 69.9% in the LVEF ≤ 50% group, *p* = 0.920). The prevalence of peripheral vascular disease, history of myocardial infarction, percutaneous intervention, coronary artery bypass grafting and complete left bundle branch block was significantly higher in patients with a reduced LVEF. These patients also more frequently used mineralocorticoid receptor antagonists (29.5% in LVEF ≤ 50% vs. 11.3% in LVEF > 50%, *p* < 0.001) and loop diuretics (91.0% in LVEF ≤ 50% vs. 76.6% in LVEF > 50%, *p* < 0.001). Checking laboratory data, median serum N-terminal pro-B-type natriuretic peptide (NT-proBNP), serum levels of creatinine, urea, C-reactive protein and hemoglobin were higher in the LVEF ≤ 50% group. The median follow-up time was 24 months (IQR 12–45 months) for those with a LVEF > 50% and 25 months (IQR 13–36) for those with a LVEF ≤ 50%. Clinical outcomes (all-cause death, 30-day death, 12-month death, cerebrovascular events, acute kidney injury, permanent pacemaker and new-onset left bundle branch block) were similar between patients with reduced and preserved LVEF. All baseline clinical characteristics and outcomes are shown in Table 1.

**Table 1:** Baseline clinical characteristics in patients with preserved (> 50%) and reduced (≤ 50%) LVEF

	Total	LVEF > 50%	LVEF ≤ 50%	*p *value
	N = 618	N = 462	N = 156	
Age (years)	82 ± 6 (50–95)	82 ± 5.4	81 ± 6.3	0.229
Male [n (%)]	291 (47.1)	193 (41.8)	98 (62.8)	< 0.001
BMI (kg/m^2^)	27.2 ± 4.8	27.3 ± 4.9	26.8 ± 4.5	0.234
NYHA class III–IV	428 (69.3)	319 (69.0)	109 (69.9)	0.920
EuroSCORE II (%)	5.0 (3.2–9.0)	5.9 ± 4.6	9.3 ± 6.1	< 0.001
Comorbidities [n (%)]				
Obesity	145 (23.5)	111 (24.0)	34 (21.8)	0.662
Hypertension	509 (82.4)	388 (84.0)	121 (77.6)	0.880
Diabetes mellitus	216 (35.0)	160 (34.6)	56 (35.9)	0.772
Dyslipidemia	387 (62.6)	281 (60.8)	106 (67.9)	0.126
Peripheral vascular disease	74 (12.0)	46 (10.0)	28 (17.9)	0.010
Chronic respiratory disease	142 (23.0)	103 (22.3)	39 (25.0)	0.510
Renal dysfunction (eGFR < 60ml/min/1.73m^2^)	365 (59.1)	270 (85.4)	95 (60.9)	0.638
History of myocardial infarction	75 (12.1)	38 (8.2)	37 (23.7)	< 0.001
History of percutaneous coronary intervention	185 (29.9)	125 (27.1)	60 (38.5)	0.009
History of CABG	71 (11.5)	35 (7.6)	36 (23.1)	< 0.001
History of atrial fibrillation	261 (42.2)	199 (43.1)	62 (39.7)	0.512
Malignancy	128 (20.7)	97 (21.0)	31 (19.9)	0.820
Complete LBBB	49 (7.9)	28 (6.1)	21 (13.5)	0.005
ICD or PM implantation	82 (13.3)	58 (12.6)	24 (15.4)	0.413
HF-related medications [n (%)]				
ACEis	298 (46.8)	217 (47.1)	81 (51.9)	0.309
ARBs	146 (23.6)	120 (26.0)	26 (16.7)	0.017
Beta-blockers	453 (73.3)	338 (73.3)	115 (73.7)	0.999
Mineralocorticoid receptor antagonists	98 (15.9)	52 (11.3)	46 (29.5)	< 0.001
Digitalis glycosides	99 (16.0)	69 (15.0)	30 (19.2)	0.209
Loop diuretics	495 (80.1)	353 (76.6)	142 (91.0)	< 0.001
Laboratory data				
Creatinine (mg/dl) [normal range 0–1.17]	1.19 (0.94–1.50)	1.15 (0.90–1.44)	1.3 (1.0–1.6)	0.002
eGFR (ml/min/1.73m^2^) [normal range > 90]	55.0 (41–71)	56.0 (41.0–71.0)	53.0 (39.3–68.0)	0.259
Urea (mg/dl) [normal range 10–50]	47.7 (36.8–66.0)	46.9 (35.2–64.5)	51.4 (40.6–72.3)	0.013
C-reactive protein (mg/dl) [normal range 0–0.5]	0.40 (0.17–1.09)	0.37 (0.14–0.96)	0.59 (0.24–1.45)	< 0.001
Hemoglobin (g/dl) [normal range 14–18]	12.3 (11.1–13.2)	12.2 (10.9–13.1)	12.6 (11.2–13.6)	0.019
Albumin (g/dl) [normal range 3.5–5.5]	4.2 (4.0–4.4)	4.2 (4.0–4.4)	4.2 (3.9–4.4)	0.453
PTT (s) [normal range 23–36]	31 (28–35)	31 (28–35)	32 (28–36)	0.486
Thrombocyte (n*1000/µl) [normal range 150–450]	220 (179–262)	221 (182–263)	216 (170–254)	0.356
Alkaline phosphatase (U/l) [normal range 40–130]	75.0 (61.0–94.0)	74.0 (61.0–92.0)	78.0 (63.0–100.0)	0.083
NT-proBNP (pg/ml)	2075 (862–6635)	1357 (634.3–3545)	6178 (1643–11,369)	< 0.001
hsTroponin (pg/ml)	36.1 (26.3–59.8)	36.2 (25.8–57.6)	34.9 (24.9–112.9)	0.802
TAVI approach				0.252
Transfemoral	385 (62.3)	294 (63.6)	91 (58.3)	
Transapical	233 (37.7)	168 (36.4)	65 (41.7)	
Clinical outcomes				
Follow-up duration (median and IQR)	24 (12–42)	24 (12–45)	25 (13–36)	0.462
All-cause death [n (%)]	279 (45.1)	206 (44.6)	73 (46.8)	0.643
30-day death [n (%)]	32 (5.2)	20 (4.3)	12 (7.7)	0.141
12-month death [n (%)]	95 (15.4)	68 (14.7)	27 (17.3)	0.443
24-month death [n (%)]	140 (22.7)	106 (22.9)	34 (21.8)	0.825
Cerebrovascular events [n (%)]	26 (4.2)	22 (4.8)	4 (2.6)	0.355
Acute kidney injury [n (%)]	58 (9.4)	41 (8.9)	17 (10.9)	0.432
Permanent pacemaker [n (%)]	47 (7.6)	34 (7.4)	13 (8.3)	0.727
New-onset LBBB [n (%)]	36 (5.8)	23 (5.0)	13 (8.3)	0.164

*P* value <0.05 is considered significant

Total number and (%) or median with (interquartile range)

ACEis, angiotensin-converting enzyme inhibitors; ARBs, angiotensin II receptor antagonists; BMI, body mass index; CAGB, Coronary artery bypass grafting; eGFR, estimated glomerular filtration rate; ICD, implantable cardioverter defibrillator; LBBB, Left bundle branch block; NYHA, New York Heart Association; PM, pacemaker; PTT, partial thromboplastin time

Baseline echocardiographic characteristics are shown in Table 2. Altogether, patients with a reduced LVEF presented signs of an enlarged right and left heart with a significantly higher left ventricular mass index (LVMi 107 g/m2 in patients with a preserved LVEF vs. 122 g/m2 in patients with a reduced LVEF, *p* < 0.001). Additionally, systolic function was reduced (TAPSE 16.7 ± 4.9 mm in LVEF ≤ 50% vs. 18.9 ± 4.7 mm in LVEF > 50%, *p* < 0.001; lateral MAPSE 8.4 ± 2.3 mm in LVEF ≤ 50% vs. 9.6 ± 2.3 mm in LVEF > 50%, *p* < 0.001; septal MAPSE 7.1 ± 1.7 mm in LVEF ≤ 50% vs. 8.2 ± 2.1 mm in LVEF > 50%, *p* < 0.001) accompanied by more severe diastolic dysfunction (E/A 1.1 in LVEF ≤ 50% vs. 0.8 in LVEF > 50%, *p* = 0.002).

**Table 2:** Baseline echocardiographic parameters in patients with preserved (> 50%) and reduced (≤ 50%) LVEF

	Total	LVEF > 50%	LVEF ≤ 50%	*p *value
	N = 618	N = 462	N = 156	
A (cm)	96 (71–119)	98 (77–122)	85 (53–109)	< 0.001
AVA Vmax (m/s)	4.2 ± 0.7	4.3 ± 0.7	3.9 ± 0.7	< 0.001
AVA PGmax (mmHg)	74 (56–87)	72 (58–90)	64 (48–78)	< 0.001
AVA PGmean (mmHg)	48 (36–57)	47 (38–59)	41 (31–50)	< 0.001
AV VTI (cm)	102.5 ± 22.6	105.2 ± 22.3	93.5 ± 21.3	< 0.001
AVA_Vmax (cm^2^)	0.99 ± 4.1	1.1 ± 4.6	0.7 ± 0.2	0.390
AVA_VTI (cm^2^)	0.78 ± 0.2	0.8 ± 0.2	0.7 ± 0.2	0.005
DT (ms)	240 (159–305)	224 (169–312)	168 (133–254)	< 0.001
E (cm/s)	99 (74–122)	97 (74–124)	95 (78–113)	0.522
E/A	1.2 (0.6–1.5)	0.8 (0.6–1.3)	1.1 (0.7–2)	0.002
E/e′ lateral	17 (11–20)	15 (11–20)	15 (12–21)	0.193
E/e′ septal	23 (16–28)	21 (15–28)	22 (17–30)	0.069
e′ lateral (cm/s)	6.5 ± 2.6	6.7 ± 2.6	6.1 ± 2.5	0.042
e′ septal (cm/s)	4.8 ± 1.8	4.9 ± 1.8	4.4 ± 1.8	0.021
IVSd (mm)	11.8 ± 2	11.9 ± 2.1	11.9 ± 2.0	0.045
LAA (cm^2^)	24.5 ± 6.8	24.2 ± 6.8	25.6 ± 6.7	0.020
LAD (mm)	42.3 ± 6.5	42.0 ± 6.4	43.3 ± 6.8	0.033
LAV (ml)	81 (59–99)	74 (57–96)	81 (63–105)	0.012
LAVi (ml/m2)	45 (32–53)	41 (32–52)	44 (34–59)	0.026
LVEDD (mm)	47.5 ± 7.6	46.4 ± 7.2	51.3 ± 7.8	< 0.001
LVEDV (ml)	84 (59–101)	72 (55–90)	106 (81–132)	< 0.001
LVESD (mm)	33.9 ± 8.5	32.2 ± 7.6	39.4 ± 8.7	< 0.001
LVESV (ml)	38 (21–48)	26 (19–35)	63 (45–81)	< 0.001
LVFS (%)	29.4 ± 10.5	31.3 ± 10.3	23.3 ± 9.0	< 0.001
LVMi (g/m2)	115 (90–135)	107 (88–133)	122 (102–144)	< 0.001
LVOT (mm)	21.9 ± 1.4	21.7 ± 1.3	22.5 ± 1.4	< 0.001
LVOT CO (l/min)	5.6 ± 1.8	5.8 ± 1.8	5.0 ± 1.5	< 0.001
LVOT PGmax (mmHg)	3 (2–4)	3 (3–4)	2 (2–3)	< 0.001
LVOT PGmean (mmHg)	1.9 ± 0.8	2.0 ± 0.8	1.4 ± 0.6	< 0.001
LVOT SV (ml)	79 (65–91)	81 (68–94)	68 (55–78)	< 0.001
LVOT Vmax (m/s)	0.84 ± 0.17	0.88 ± 0.2	0.73 ± 0.2	< 0.001
LVOT VTI (cm)	20.9 ± 5.1	22.1 ± 4.8	17.2 ± 4.3	< 0.001
LVPWd (mm)	11.4 ± 1.9	11.4 ± 1.9	11.1 ± 1.8	0.052
MAPSE lateral (mm)	9.3 ± 2.3	9.6 ± 2.3	8.4 ± 2.3	< 0.001
MAPSE septal (mm)	7.9 ± 2.1	8.2 ± 2.1	7.1 ± 1.7	< 0.001
RAA (cm^2^)	18 (13–21)	17 (13–21)	17 (14–24)	0.019
RVD basal (mm)	36.8 ± 7	36.2 ± 6.7	38.7 ± 7.9	0.002
RVD mid (mm)	27.7 ± 6.6	27.3 ± 6.2	29.0 ± 7.7	0.021
sPAP (mmHg)	41 (32–49)	39 (32–48)	41 (32–50)	0.533
TAPSE (mm)	18.4 ± 4.9	18.9 ± 4.7	16.7 ± 4.9	< 0.001
TR PGmax (mmHg)	36 (27–42)	34 (27–42)	35 (27–44)	0.540
TR Vmax (m/s)	2.9 ± 0.6	2.9 ± 0.6	2.9 ± 0.6	0.996

*P* value <0.05 is considered significant

Mean ± standard deviation or median with (interquartil range)

A, pulsed-wave Doppler derived late diastolic mitral inflow velocity; AVA Vmax, peak velocity in aortic valve area; AVA PGmax, maximum gradient in aortic valve area; AVA PGmean, mean gradient in aortic valve area; AVA VTI, velocity–time integral of aortic valve; AVA: aortic valve area; DT, deceleration time of the mitral E wave; E, pulsed-wave Doppler derived early diastolic mitral inflow velocity; E/A ratio, the ratio of early to late diastolic filling velocity; E/e′ ratio, the ratio of early diastolic filling velocity to mitral annular velocity; e´, early diastolic filling velocity; IVSd, interventricular septum wall thickness at end-diastole; LAA, area of left atrium; LAD, diameter of left atrium; LAV, volume of left atrium; LAVi, left atrial volume index at end-systole; LVEDD, left ventricular dimension at end-diastole; LVEDV, left ventricular volume at end-diastole; LVESD, left ventricular dimension at end-systole; LVESV, left ventricular volume at end-systole; LVFS, left ventricular fractional shortening; LVMi, left ventricular mass index; LVOT, left ventricular outflow tract; LVOT CO, left ventricular outflow tract cardiac output; LVOT PGmax, left ventricular outflow tract maximum gradient; LVOT PGmean, left ventricular outflow tract mean gradient; LVOT SV, left ventricular outflow tract stroke volume; LVOT Vmax, left ventricular outflow tract peak velocity; LVOT VTI, left ventricular outflow tract velocity–time-integral; LVPWd, left ventricular posterior wall thickness at end-diastole; MAPSE, mitral annular plane systolic excursion; RAA, right atrial area at end-systole; RVD, right ventricular diameter at end-diastole; sPAP, systolic pulmonary artery pressure; TAPSE, tricuspid annular plane systolic excursion; TR PGmax; maximum gradient of tricuspid valva; TRVmax, peak tricuspid regurgitation jet velocity

### Survival and left ventricular ejection fraction

**Fig. 2:**
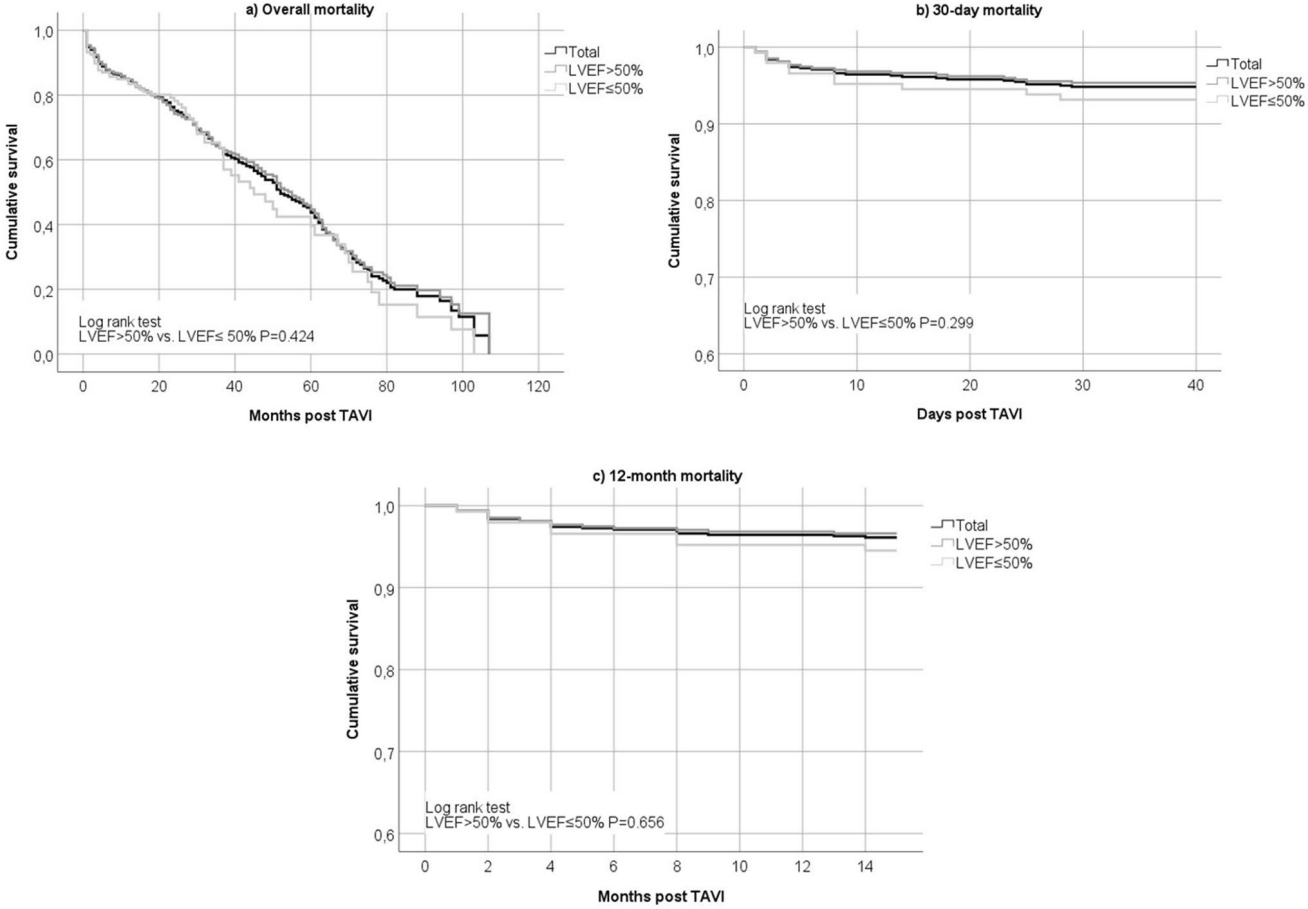
Survival curves displaying overall (**a**), short-term (**b**) and long-term (**c**) mortality after TAVI in patients with preserved (> 50%) and reduced (≤ 50%) LVEF

The mean survival time post TAVI was 51 months [95% CI (48–55)]. Survival time was similar between patients with preserved and reduced LVEF in terms of overall mortality rate (log rank test LVEF > 50% vs. LVEF ≤ 50% *p* = 0.424), at 30 days (log rank test LVEF > 50% vs. LVEF ≤ 50% *p* = 0.299) and 12 months post TAVI (log rank test LVEF > 50% vs. LVEF ≤ 50% *p* = 0.656). Survival curves are displayed in Fig. 2.

### Clinical risk factors associated with all-cause death overall mortality, 30-day mortality and 12-month mortality

After univariate Cox regression (LVEF > 50% Table 3, LVEF ≤ 50% Table 4), potential clinical covariates were evaluated in a multivariate Cox regression model (Table 5) showing that overall mortality in patients with a preserved LVEF is predicted by BMI (HR 0.958, 95% CI 0.929–0.988, *p* = 0.006), transapical TAVI approach (HR 1.710, 95% CI 1.280–2.286, *p* < 0.001), dyslipidaemia (HR 1.335, 95% CI 1.017–1.754, *p* = 0.037) and C-reactive protein levels (HR 1.139, 95% CI 1.014–1.279, *p* = 0.028). Clinical predictors for 30-day mortality were male sex (HR 3.521, 95% CI 1.189–10.24, *p* = 0.023), BMI (HR 0.853, 95% CI 0.772–0.941, *p* = 0.002), C-reactive protein levels (HR 1.819, 95% CI 1.317–2.512, *p* < 0.001) and use of amiodarone (HR 10.532, 95% CI 2.895–38.316, *p* < 0.001). Twelve-month mortality presented similar trends. In patients with a reduced LVEF, fewer covariates could be detected: NYHA class III-IV (HR 2.429, 95% CI 1.277–4.620, *p* = 0.007), C-reactive protein levels (HR 1.497, 95% CI 1.184–1.894, *p* < 0.001) and hemoglobin levels (HR 0.848, 95% CI 0.729–0.987, *p* = 0.033) for overall mortality; C-reactive protein levels (HR 1.950, 95% CI 1.359–2.797, *p* < 0.001) and use of antiplatelet drugs (HR 0.354, 95% CI 0.149–0.842, *p* = 0.019) for 12-month mortality. No clinical predictors could be determined for 30-day mortality.

**Table 3:** Univariate Cox Regression for LVEF > 50% and clinical risk factors associated with all-cause death at overall mortality, 30-day mortality and 12-month mortality

	Overall mortality			30-day mortality			12-month mortality		
	Unadjusted HR	95% CI	*p* value	Unadjusted HR	95% CI	*p* value	Unadjusted HR	95% CI	*p* value
Age (years)	1.000	0.973–1.028	0.993	1.021	0.940–1.109	0.620	0.992	0.950–1.036	0.707
Sex	0.880	0.669–1.158	0.362	3.299	1.117–9.749	0.031	1.136	0.706–1.828	0.600
BMI (kg/m2)	0.969	0.941–0.998	0.039	0.849	0.763–0.945	0.003	0.940	0.891–0.992	0.023
TAVI approach transapical versus transfemoral	1.810	1.370–2.393	< 0.001	2.035	0.879–4.710	0.097	1.933	1.214–3.080	0.006
CKD stage 3–5	1.434	1.084–1.898	0.012	1.897	0.742–4.849	0.181	1.521	0.925–2.500	0.098
NYHA III-IV	1.073	0.801–1.437	0.636	0.787	0.330–1.875	0.588	1.135	0.677–1.904	0.631
Dyslipidemia	1.335	1.018–1.750	0.037	1.572	0.682–3.626	0.289	1.484	0.932–2.365	0.096
Chronic respiratory disease	1.151	0.840–1.576	0.383	1.049	0.387–2.843	0.925	0.957	0.542–1.693	0.881
Peripheral vascular disease	1.334	0.896–1.986	0.155	1.404	0.415–4.743	0.585	1.690	0.888–3.214	0.110
Atrial fibrillation	1.347	1.029–1.763	0.030	1.622	0.701–3.753	0.259	1.717	1.075–2.740	0.024
Hypertension	1.108	0.757–1.622	0.597	0.840	0.284–2.482	0.753	1.162	0.596–2.269	0.659
Diabetes mellitus	1.017	0.768–1.345	0.908	1.081	0.453–2.577	0.861	1.024	0.629–1.667	0.923
Myocardial infarction	1.207	0.782–1.860	0.395	0.043	0.001–27.823	0.341	0.591	0.216–1.622	0.307
Creatinine (mg/dl)	1.051	1.011–1.092	0.011	1.034	0.896–1.194	0.647	1.040	0.969–1.117	0.277
Urea (mg/dl)	1.008	1.004–1.012	< 0.001	1.000	0.986–1.013	0.968	1.006	0.999–1.012	0.081
C-reactive protein (mg/dl)	1.225	1.096–1.369	< 0.001	1.667	1.201–2.312	0.002	1.454	1.211–1.746	< 0.001
Hemoglobin (g/dl)	0.870	0.802–0.944	< 0.001	0.788	0.617–1.006	0.056	0.802	0.700–0.919	0.001
LDL-Cholesterin (mg/dl)	0.996	0.992–1.000	0.070	1.000	0.986–1.015	0.968	0.996	0.989–1.004	0.358
Use of amiodarone	1.975	0.973–4.010	0.059	7.262	2.147–24.565	0.001	3.838	1.546–9.528	0.004
Use of antiplatelet drugs	0.962	0.735–1.260	0.778	0.790	0.343–1.823	0.581	0.721	0.452–1.148	0.168
*HF medications*									
Mineralcorticoid receptor antagonist	1.173	0.777–1.769	0.447	0.041	0.001–13.489	0.281	1.162	0.577–2.337	0.674
Digitalis Glykosid	1.054	0.742–1.499	0.768	0.907	0.268–3.065	0.875	1.048	0.551–1.993	0.887
Betablocker	1.192	0.878–1.619	0.259	1.643	0.556–4.854	0.369	1.383	0.782–2.445	0.265
Loop diuretics	1.406	0.993–1.990	0.055	1.372	0.464–4.056	0.567	1.351	0.740–2.466	0.327
ACE inhibitors	0.939	0.716–1.230	0.646	1.136	0.493–2.620	0.765	1.189	0.747–1.894	0.465
ARBs	0.834	0.606–1.148	0.266	0.441	0.131–1.492	0.188	0.667	0.372–1.197	0.175

Hazard ratio (HR) with 95% confidence interval (CI)

ACEis, angiotensin-converting enzyme inhibitors; ARBs, angiotensin II receptor antagonists; BMI, body mass index; CKD: chronic kidney disease; HF: heart failure; NYHA: New York Heart Association

**Table 4:** Univariate Cox Regression for LVEF ≤ 50% and clinical risk factors associated with all-cause death at overall mortality, 30-day mortality and 12-month mortality

	Overall mortality			30-day mortality			12-month mortality		
	Unadjusted HR	95% CI	*p* value	Unadjusted HR	95% CI	*p* value	Unadjusted HR	95% CI	*p* value
Age (years)	0.996	0.959–1.035	0.848	1.027	0.925–1.142	0.614	0.986	0.929–1.046	0.629
Sex	0.808	0.478–1.366	0.426	0.446	0.095–2.102	0.307	0.578	0.229–1.457	0.245
BMI (kg/m2)	0.980	0.925–1.038	0.493	0.874	0.743–1.029	0.106	0.958	0.870–1.054	0.380
TAVI approach transapical versus transfemoral	1.361	0.820–2.258	0.233	1.541	0.446–5.325	0.494	1.331	0.596–2.972	0.485
CKD stage 3–5	0.789	0.472–1.317	0.365	0.645	0.187–2.228	0.488	0.441	0.196–0.994	0.056
NYHA III-IV	1.893	1.038–3.455	0.037	0.612	0.173–2.169	0.447	0.971	0.403–2.342	0.948
Dyslipidemia	0.814	0.479–1.384	0.448	0.514	0.109–2.420	0.400	0.993	0.425–2.320	0.987
Chronic respiratory disease	1.434	0.836–2.462	0.191	0.713	0.151–3.359	0.669	0.939	0.373–2.365	0.894
Peripheral vascular disease	1.731	0.949–3.159	0.074	1.168	0.248–5.502	0.844	1.596	0.634–4.022	0.321
Atrial fibrillation	1.172	0.704–1.950	0.542	0.989	0.279–3.505	0.986	1.536	0.690–3.420	0.293
Hypertension	1.231	0.681–2.228	0.491	0.430	0.121–1.523	0.191	0.856	0.340–2.157	0.742
Diabetes mellitus	1.377	0.813–2.331	0.234	1.198	0.338–4.245	0.780	0.745	0.309–1.796	0.511
Myocardial infarction	1.267	0.715–2.244	0.418	1.442	0.373–5.577	0.596	1.172	0.465–2.954	0.736
Creatinine (mg/dl)	1.188	0.914–1.545	0.198	1.407	0.992–1.996	0.056	1.064	0.692–1.634	0.778
Urea (mg/dl)	1.009	1.000–1.018	0.056	1.016	0.997–1.036	0.102	0.995	0.978–1.012	0.552
C-reactive protein (mg/dl)	1.513	1.210–1.893	< 0.001	1.728	1.083–2.755	0.022	2.088	1.504–2.898	< 0.001
Hemoglobin (g/dl)	0.825	0.705–0.966	0.017	0.962	0.669–1.384	0.836	0.764	0.600–0.972	0.028
LDL-Cholesterin (mg/dl)	1.001	0.994–1.007	0.856	1.002	0.985–1.019	0.831	0.997	0.986–1.009	0.661
Use of amiodarone	1.201	0.369–3.905	0.761	2.758	0.349–21.781	0.336	2.396	0.563–10.198	0.237
Use of antiplatelet drugs	0.578	0.347–0.963	0.035	0.285	0.074–1.103	0.069	0.308	0.132–0.720	0.007
*HF medications*									
Mineralcorticoid receptor antagonist	0.968	0.552–1.698	0.910	0.541	0.115–2.549	0.437	1.076	0.460–2.514	0.866
Digitalis Glykosides	1.595	0.901–2.823	0.109	1.012	0.215–4.764	0.988	2.146	0.918–5.016	0.078
Betablocker	1.801	0.635–1.840	0.774	0.547	0.154–1.939	0.350	0.731	0.313–1.709	0.470
Loop diuretics	3.062	0.951–9.856	0.061	0.901	0.114–7.111	0.921	2.238	0.302–16.579	0.430
ACE inhibitors	0.856	0.520–1.408	0.539	0.866	0.251–2.990	0.819	0.603	0.268–1.358	0.222
ARBs	0.917	0.452–1.862	0.811	0.038	0.001–55.36	0.379	0.465	0.109–1.976	0.299

Hazard ratio (HR) with 95% confidence interval (CI)

ACEis, angiotensin-converting enzyme inhibitors; ARBs, angiotensin II receptor antagonists; BMI, body mass index; CKD: chronic kidney disease; HF: heart failure; NYHA: New York Heart Association

**Table 5:** Multivariate Cox Regression for clinical risk factors associated with all-cause death at overall mortality, 30-day mortality and 12-month mortality

	Overall mortality			30-day mortality			12-month mortality		
	Clinical covariates adjusted HR	95% CI	*p* value	Clinical covariates adjusted HR	95% CI	*p* value	Clinical covariates adjusted HR	95% CI	*p* value
*LVEF* > *50%*									
Sex	–	–	–	3.521	1.189–10.424	0.023	–	–	–
BMI (kg/m2)	0.958	0.929–0.988	0.006	0.853	0.772–0.941	0.002	0.936	0.889–0.986	0.013
TAVI approach transapical versus transfemoral	1.710	1.280–2.286	< 0.001	–	–	–	1.687	1.043–2.731	0.033
CKD stage 3–5	1.189	0.849–1.665	0.314	–	–	–	–	–	–
Dyslipidemia	1.335	1.017–1.754	0.037	–	–	–	–	–	–
Atrial fibrillation	1.167	0.887–1.536	0.271	–	–	–	1.345	0.827–2.187	0.232
Creatinine (mg/dl)	1.042	0.983–1.105	0.165	–	–	–	–	–	–
Urea (mg/dl)	1.004	0.998–1.009	0.167	–	–	–	–	–	–
C-reactive protein (mg/dl)	1.139	1.014–1.279	0.028	1.819	1.317–2.512	< 0.001	1.365	1.125–1.657	0.002
Hemoglobin (g/dl)	0.914	0.832–1.003	0.057	–	–	–	0.868	0.750–1.004	0.057
Use of amiodarone	–	–	–	10.532	2.895–38.316	< 0.001	3.266	1.132–9.424	0.029
*LVEF* ≤ *50%*									
NYHA III-IV	2.429	1.277–4.620	0.007	–	–	–	–	–	–
C-reactive protein (mg/dl)	1.497	1.184–1.894	< 0.001	–	–	–	1.950	1.359–2.797	< 0.001
Hemoglobin (g/dl)	0.848	0.729–0.987	0.033	–	–	–	0.883	0.684–1.140	0.341
Use of antiplatelet drugs	0.610	0.358–1.040	0.069	–	–	–	0.354	0.149–0.842	0.019

Hazard ratio (HR) with 95% confidence interval (CI)

BMI, body mass index; CKD: chronic kidney disease; NYHA: New York Heart Association

### Independent echocardiographic predictors for TAVI patients with preserved or reduced LVEF

Univariate Cox regression models for echocardiographic predictors exposed several potential risk factors for patients with preserved and reduced LVEF. Left ventricular systolic function, however, did not remain significant (Table 6).

**Table 6:** Univariate Cox Regression for echocardiographic risk factors associated with all-cause death at overall mortality, 30-day mortality and 12-month mortality

	Overall mortality			30-day mortality			12-month mortality		
	Univariate HR	95% CI	*p* value	Univariate HR	95% CI	*p* value	Univariate HR	95% CI	*p* value
**LVEF > 50%**									
Cardiac Sizes									
IVSd (mm)	1.020	0.954–1.090	0.567	1.244	1.059–1.461	0.008	1.065	0.954–1.190	0.263
LAA (cm^2^)	1.028	1.008–1.048	0.005	1.054	1.001–1.109	0.046	1.028	0.996–1.061	0.091
LAD (mm)	1.031	1.009–1.053	0.006	1.063	0.994–1.137	0.076	1.047	1.009–1.086	0.014
LVEDD (mm)	0.999	0.980–1.018	0.900	0.951	0.896–1.009	0.095	0.975	0.944–1.007	0.128
LVMi (g/m2)	1.002	0.998–1.005	0.399	1.004	0.993–1.016	0.452	0.999	0.993–1.006	0.859
LVPWd (mm)	1.026	0.956–1.100	0.478	1.235	1.028–1.483	0.024	1.047	0.928–1.181	0.457
RAA (cm^2^)	1.024	1.005–1.044	0.014	1.032	0.976–1.090	0.271	1.029	0.997–1.062	0.077
RVD_basal (mm)	1.021	0.999–1.044	0.061	1.010	0.943–1.082	0.772	1.008	0.971–1.046	0.677
RVD_mid (mm)	1.017	0.993–1.041	0.177	1.005	0.933–1.082	0.905	1.004	0.965–1.044	0.844
LVEF (%)	0.991	0.973–1.010	0.352	0.990	0.937–1.046	0.728	0.978	0.948–1.009	0.165
Systolic function									
MAPSE lateral (mm)	0.918	0.864–0.975	0.006	0.862	0.695–1.070	0.177	0.917	0.821–1.025	0.126
MAPSE septal (mm)	0.883	0.823–0.947	< 0.001	0.708	0.545–0.921	0.010	0.836	0.739–0.947	0.005
TAPSE (mm)	0.949	0.920–0.979	< 0.001	0.888	0.801–0.985	0.025	0.928	0.880–0.978	0.006
DD									
Moderate to severe versus mild	1.445	1.089–1.919	0.011	3.179	1.076–9.394	0.036	1.861	1.110–3.121	0.019
Lateral E/E′	1.003	0.986–1.020	0.770	1.028	0.985–1.072	0.205	0.999	0.970–1.029	0.957
Septal E/E′	1.017	1.003–1.031	0.015	1.062	1.026–1.098	< 0.001	1.028	1.006–1.051	0.013
sPAP (mmHg)	1.020	1.011–1.030	< 0.001	1.042	1.016–1.068	0.001	1.025	1.008–1.041	0.003
**LVEF≤50%**									
Cardiac Sizes									
IVSd (mm)	1.059	0.934–1.200	0.372	0.845	0.617–1.158	0.295	0.948	0.776–1.158	0.602
LAA (cm^2^)	1.022	0.984–1.062	0.266	1.027	0.932–1.133	0.586	1.057	0.994–1.124	0.076
LAD (mm)	1.032	0.995–1.070	0.093	1.011	0.924–1.107	0.806	1.060	1.001–1.123	0.045
LVEDD (mm)	0.980	0.953–1.009	0.173	1.014	0.936–1.098	0.741	1.000	0.951–1.052	0.999
LVMi (g/m2)	0.997	0.990–1.005	0.507	0.994	0.975–1.013	0.540	1.001	0.989–1.014	0.827
LVPWd (mm)	1.103	0.948–1.282	0.204	0.849	0.595–1.212	0.368	1.120	0.897–1.399	0.317
RAA (cm^2^)	1.035	0.994–1.079	0.099	1.051	0.953–1.160	0.318	1.060	0.998–1.126	0.060
RVD_basal (mm)	0.993	0.958–1.029	0.701	0.939	0.845–1.042	0.236	0.968	0.913–1.026	0.269
RVD_mid (mm)	0.994	0.959–1.031	0.749	0.927	0.828–1.036	0.182	0.956	0.897–1.018	0.159
LVEF (%)	1.001	0.966–1.037	0.954	0.978	0.913–1.048	0.533	1.027	0.971–1.086	0.354
Systolic function									
MAPSE lateral (mm)	1.001	0.898–1.115	0.989	1.005	0.755–1.338	0.971	1.053	0.890–1.246	0.545
MAPSE septal (mm)	0.849	0.723–0.997	0.047	1.162	0.813–1.662	0.410	0.939	0.736–1.198	0.613
TAPSE (mm)	0.951	0.899–1.006	0.079	0.953	0.820–1.107	0.526	0.957	0.875–1.048	0.343
DD									
Moderate to severe versus mild	1.130	0.653–1.955	0.663	1.550	0.329–7.300	0.579	1.190	0.472–2.998	0.712
Lateral E/E′	0.988	0.961–1.015	0.379	1.030	0.978–1.085	0.263	0.986	0.938–1.037	0.580
Septal E/E′	1.009	0.988–1.030	0.409	1.031	0.974–1.091	0.290	1.012	0.973–1.051	0.554
sPAP (mmHg)	1.001	0.983–1.019	0.946	1.006	0.958–1.057	0.801	1.010	0.980–1.040	0.521

Hazard ratio (HR) with 95% confidence interval (CI)

DD: diastolic dysfunction; E/e′ ratio, the ratio of early diastolic filling velocity to mitral annular velocity; e´, early diastolic filling velocity; IVSd, interventricular septum wall thickness at end-diastole; LAA, area of left atrium; LAD, diameter of left atrium; LVEDD, left ventricular dimension at end-diastole; LVMi, left ventricular mass index; LVPWd, left ventricular posterior wall thickness at end-diastole; MAPSE, mitral annular plane systolic excursion; RAA, right atrial area at end-systole; RVD, right ventricular diameter at end-diastole; sPAP, systolic pulmonary artery pressure; TAPSE, tricuspid annular plane systolic excursion

Multivariate Cox regression revealed following echocardiographic parameters as risk factors in patients with preserved LVEF: TAPSE, sPAP and septal E/E′. In that lower TAPSE (≤ 17 vs. > 17 mm, HR 1.528, 95% CI 1.083–2.154, *p* = 0.016) and higher sPAP (> 30 vs. ≤ 30 mmHg, HR 1.900, 95% CI 1.253–2.880, *p* = 0.002) remained as independent predictors for overall mortality; higher septal E/E′ remained as independent predictor for 30-day (> 21 vs. ≤ 21, HR 14.462, 95% CI 1.892–110.550, *p* = 0.010) and 12-month (> 21 vs. ≤ 21, HR 1.881, 95% CI 1.079–3.278, *p* = 0.026) mortality.

No independent echocardiographic predictors regarding mortality in TAVI patients with a LVEF ≤ 50% could be determined (Table 7).

**Table 7:** Multivariate Cox regression for echocardiographic predictors. Into the final model significant echocardiographic and clinical predictors were included

	Multivariable model including echocardiographic covariates		Final model including echocardiographic and clinical covariates	
Overall mortality: echocardiographic predictors for patients with LVEF > 50%				
	HR (95% CI)	*p* value	HR (95% CI)	*p* value
LAA (cm^2^)	1.013 (0.983–1.045)	0.393		
RAA (cm^2^)	0.992 (0.962–1.024)	0.627		
MAPSE septal (mm)	0.944 (0.862–1.033)	0.206		
TAPSE (mm)	0.958 (0.922–0.996)	0.031	0.951 (0.921–0.982)	0.002
≤ 17 versus > 17 mm			1.528 (1.083–2.154)	0.016
DD moderate to severe versus mild	0.813 (0.539–1.226)	0.323		
E/E′ septal	1.011 (0.995–1.027)	0.176		
sPAP (mmHg)	1.015 (1.003–1.028)	0.014	1.015 (1.004–1.025)	0.007
> 30 versus ≤ 30 mmHg			1.900 (1.253–2.880)	0.002

Overall mortality: Echocardiographic predictors for patients with LVEF ≤ 50%				
	HR (95% CI)	*p* value	HR (95% CI)	*p* value
MAPSE septal (mm)			0.883 (0.751–1.037)	0.129

30-day mortality: echocardiographic predictors for patients with LVEF > 50%				
	HR (95% CI)	*p* value	HR (95% CI)	*p* value
IVSd (mm)	1.085 (0.866–1.360)	0.478		
LAA (cm^2^)	1.036 (0.954–1.126)	0.398		
MAPSE septal (mm)	0.933 (0.672–1.295)	0.679		
TAPSE (mm)	0.924 (0.809–1.055)	0.244		
DD moderate to severe versus mild	0.773 (0.167–3.578)	0.742		
E/E′ septal	1.048 (1.003–1.095)	0.035	1.072 (1.031–1.114)	< 0.001
> 21 versus ≤ 21			14.462 (1.892–110.550)	0.010
sPAP (mmHg)	1.019 (0.979–1.061)	0.354		

30-day mortality: echocardiographic predictors for patients with LVEF ≤ 50%				
	HR (95% CI)	*p* value	HR (95% CI)	*p* value
–	–	–	–	–

12-month mortality: Echocardiographic predictors for patients with LVEF > 50%				
	HR (95% CI)	*p* value	HR (95% CI)	*p* value
LAD (mm)	1.030 (0.982–1.080)	0.230		
MAPSE septal (mm)	0.942 (0.808–1.099)	0.450		
TAPSE (mm)	0.966 (0.906–1.031)	0.301		
DD moderate to severe versus mild	0.993 (0.499–1.971)	0.983		
E/E′ septal	1.027 (1.002–1.052)	0.036	1.027 (1.004–1.050)	0.019
> 21 versus ≤ 21			1.881 (1.079–3.278)	0.026
sPAP (mmHg)	1.002 (0.981–1.024)	0.853		

12-month mortality: Echocardiographic predictors for patients with LVEF ≤ 50%				
	HR (95% CI)	*p* value	HR (95% CI)	*p* value
LAD (mm)			1.011 (0.951–1.074)	0.726

Hazard ratio (HR) with 95% confidence interval (CI)

DD: diastolic dysfunction; E/e′ ratio, the ratio of early diastolic filling velocity to mitral annular velocity; e´, early diastolic filling velocity; IVSd, interventricular septum wall thickness at end-diastole; LAA, area of left atrium; LAD, diameter of left atrium; MAPSE, mitral annular plane systolic excursion; sPAP, systolic pulmonary artery pressure; TAPSE, tricuspid annular plane systolic excursion

Survival curves displaying the impact of independent echocardiographic predictors are evaluated in Kaplan–Meier curves in the Supplement Fig. S2. Results showed that survival in patients with preserved LVEF and aforementioned risk factors is significantly poorer: TAPSE (log rank test ≤ 17 mm vs. > 17 mm *p* = 0.024) and sPAP (log rank test > 30 mmHg vs. ≤ 30 mmHg *p* < 0.001) for overall mortality; E/E′ septal for 30-day mortality (log rank test > 21 vs. ≤ 21 *p* < 0.001) and 12-month mortality (log rank test > 21 vs. ≤ 21 *p* = 0.023).

TAVI approach is a known impact factor on outcome. We explored subgroup analysis to define the potential echocardiographic predictors on patients with preserved or reduced LVEF receiving various TAVI approaches: LVEF > 50% or LVEF ≤ 50% receiving transfemoral or transapical approach. Significant differences in survival between the groups could be observed (Supplement Table S1 and Fig. S1), especially between the group LVEF > 50% + transfemoral approach versus the group LVEF > 50% + transapical approach (log rank test *p* < 0.001). No differences are found between the groups with reduced LVEF receiving transfemoral or transapical approach (log rank test *p* = 0.227). To evaluate possible echocardiographic predictors on outcome for TAVI patients with preserved or reduced LVEF receiving various approaches, additional univariate/multivariate Cox regression analyses within each subgroup (Supplement Table S2) were performed. Results showed that in the subgroup LVEF > 50% + transfemoral approach, multivariate Cox regression after checking for collinearity revealed that septal MAPSE (HR 0.852, 95% CI 0.740–0.982, *p* = 0.027) and sPAP (HR 1.021, 95% CI 1.004–1.039, *p* = 0.015) are independent predictors for increased overall mortality post TAVI. TAPSE (HR 0.896, 95% CI 0.813–0.988, *p* = 0.027) remained as a risk factor for increased postprocedural mortality in the group LVEF ≤ 50% + transapical approach.

## Discussion

The main findings of this study are as follows: (i) Cardiovascular comorbidities such as dyslipidaemia and pulmonary hypertension (reflected as increased sPAP) imply a higher risk of death in TAVI patients postprocedurally. (ii) Independent echocardiographic predictors for survival post TAVI are lower TAPSE, higher sPAP and higher septal E/E′. (iii) LVEF is not a predictor of short- and long-term mortality after TAVI.

Although patients with a reduced ejection fraction generally have poorer outcomes [9], this does not adversely affect outcome in patients undergoing TAVI. Not only was mortality (at 30 days, 12 months and overall) similar between patients with reduced and preserved LVEF, but the frequency of TAVI-specific complications (cerebrovascular events, acute kidney injury, etc.) was also similar. Furthermore, after adjustment for clinical risk factors, no echocardiographic predictor could be identified for TAVI patients with a LVEF ≤ 50%. Our real-world data confirm results from randomized clinical trials, such as the PARTNER Trial [10], stating that LVEF does not impact survival postprocedurally. In line with recent literature, we additionally identified multiple clinical and echocardiographic parameters associated with survival post TAVI. Most prominently, elevated systolic pulmonary artery pressure (sPAP) seems to have a significant impact on survival postprocedurally [11, 12]. Vizzardi et al. already suggested careful evaluation of right ventricular deformation in TAVI patients with heart failure [13]. We agree that right heart function should be examined and that the results should be taken into account very carefully prior to TAVI. Additionally, our results show that lower TAPSE also represents a higher risk for mortality, suggesting reduced longitudinal systolic function again focusing on the right heart. These findings are in line with the results of prior studies [14]. The recently published meta-analysis by Stens et al. emphasizes the importance of reduced LV global longitudinal strain on survival post TAVI and the risk for major cardiovascular events [15]. Since strain measurements depend on high image quality [16, 17], we suggest that the parameters MAPSE and TAPSE serve as alternative parameters for risk stratification when strain measurement is not available (e.g. poor image quality due to patients’ factors or technical equipment). Regular evaluation of both left and right ventricular systolic function currently plays a key role in the first detection of changes in cardiac function, largely affecting further treatment [18, 19]. Here, while myocardial contractility is central to survival, diastolic function with impaired relaxation is also important. Higher septal left ventricular filling pressure E/E′ especially affects short-term outcomes in TAVI patients with a preserved LVEF. The importance of advanced diastolic dysfunction and its effect on survival post TAVI is described in the literature [20, 21]. We sought to find a cut-off value to simplify the identification of patients at higher risk. The echocardiographic parameters identified serve as signs of structural changes in the heart, which might result from aortic valve impairment, cardiovascular comorbidities, or a combination of both. We see here a connection between the clinical and echocardiographic predictors, resulting from or causing deterioration of the other parameters and therefore effecting a circulus vitiosus. Regarding the clinical factors, we suggest that arrhythmias negatively affect survival. While atrial fibrillation itself could not be determined as a relevant risk factor in multivariate regression, the use of amiodarone reflected an indirect hint. TAVI patients taking amiodarone were approximately ten times as likely to die in the short term, representing the most powerful risk factor we found in our current study. As described in the 2020 ESC guidelines, amiodarone currently represents the best option for long-term rhythm control in patients with valvular disease [22]. Therefore, the persistence of preinterventional arrhythmias suggests a poorer outcome post TAVI. We also found signs of systemic involvement in patients with aortic valve stenosis, as indicated by higher levels of C-reactive protein. Increased levels was the only risk factor identified for TAVI patients with preserved and reduced LVEF as well as overall outcomes. While the detailed pathogenesis of aortic stenosis is still unclear, higher C-reactive protein as an acute phase protein may indicate a more active state of inflammation being part of the pathogenesis [23], resulting in a possible higher cardiovascular risk profile.

We assume that the reasons for higher mortality in these patients are not linked to (global) heart function but rather to cardiovascular complications (e.g. atrial fibrillation with embolism or bleeding due to anticoagulant use) on one hand and right heart pathologies (decreased TAPSE, increased sPAP) on the other hand. Raju et al. postulated that vascular complications affect operative morbidity in TAVI patients [24], which ultimately can result in an increased mortality.

Intervention of the aortic valve may change the natural course of heart failure by impacting at least some of the underlying mechanisms (modelling, myocardial fibrosis, hypertrophy) in a positive way. Looking at the results of the study, we therefore assume that respective positive effects account for the leveling up of patients with preserved and reduced LVEF after TAVI. As the features mentioned above indicate a higher risk of death post TAVI in patients with a preserved LVEF, we suggest carefully assessing them preinterventionally and managing this “high-risk” group particularly closely. We see hints that especially right heart function/dysfunction has an impact on survival post TAVI and not (only) left heart pathologies caused by aortic valve calcification. Postinterventional check ups should therefore not only focus on the valve prothesis and the global heart function but examine more closely right heart function. This not only relates to preinterventional risk stratification but might also contribute to a more individualized and/or intensified treatment both pre- and postintervention. Future studies are needed to observe whether intensive perioperative monitoring of these high-risk patients could improve their post-TAVI outcome.

### Clinical implication

We identified the preinterventional signs of myocardial and extracardiac alterations that affected postintervention survival in TAVI patients. Strikingly, left ventricular ejection fraction is not a predictor of increased mortality, as clinical outcomes between TAVI patients with reduced LVEF (≤ 50%) and preserved LVEF (> 50%) are similar, and no echocardiographic predictor for a worse outcome could be determined for TAVI patients with a reduced LVEF. We recommend to pay enhanced attention to high-risk TAVI patients presenting with aforementioned cardiovascular risk profile and echocardiographic predictors during follow-up. Future prospective clinical study is warranted to verify this concept.

### Limitations

Echocardiographic variables were extracted from transthoracic echocardiograms (TTEs) clinically recorded prior to TAVI. Since echocardiographic measurements largely depend on the experience and practice of the investigators, we cannot rule out deviations in measurements or calculation of echocardiographic parameters, especially due to the long study duration and high number of investigators involved. Furthermore, we divided our cohort into several subgroups, creating different unequal group sizes, which might affect the results. With regard to the study outcomes, we could not identify the actual causes of patient deaths, i.e., differentiating cardiac from noncardiac death, due to the retrospective nature of the study.

## Conclusions

LVEF is not a predictor of short- or long-term mortality after TAVI. Therefore, a reduced LVEF should not prevent patients from undergoing TAVI. Left ventricular filling pressure (E/E´), systolic pulmonary artery pressure (sPAP) and TAPSE represent clinically relevant measures to determine mortality risk post TAVI in patients with a preserved LVEF. Relevant non echocardiographic clinical predictors of poor outcome after TAVI include atrial fibrillation and elevated CRP levels.

### Supplementary Information

Below is the link to the electronic supplementary material.

Supplementary file 1 (DOCX 297 KB)

## Data Availability

The authors confirm that the data supporting the findings of this study are available within the article and its supplementary materials. Data sharing is not applicable in this study due to the restrictions of local ethical review boards.
