# Supplementary material for: Echocardiographic predictors of outcome in severe aortic stenosis patients with preserved or reduced ejection fraction
Source: Clin Res Cardiol. 2024 Jan 22;113(3):481–95. doi: 10.1007/s00392-023-02350-w (PMC10881626; doi:10.1007/s00392-023-02350-w)
Supplement: Supplementary file 1 — (DOCX 297 KB) [file 392_2023_2350_MOESM1_ESM.docx]

**Supplement**

|  | LVEF>50%+TF  N=297 | LVEF≤50%+TF  N=88 | LVEF>50%+TA  N=175 |  | LVEF>50%+TA  N=58 | p-value |
| --- | --- | --- | --- | --- | --- | --- |
| Age (years)  Male [n (%)]  BMI (kg/m²)  NYHA class III-IV  Comorbidities [n (%)]  Obesity  Hypertension  Diabetes  Dyslipidemia  Renal dysfunction (eGFR<60ml/min/1.73m²)  History of atrial fibrillation    Laboratory data  Creatinine (mg/dl) [normal range 0-1.17]  eGFR (ml/min/1.73m²) [normal range >90]  Urea (mg/dl) [normal range 10-50]  C-reactive protein (mg/dl) [normal range 0-0.5]  Hemoglobin (g/dl) [normal range 14-18]  NT-proBNP (pg/ml)  hsTroponin (pg/ml)    Clinical outcomes  All-cause death [n (%)]  30-day death [n (%)]  12-month death [n (%)]  Cerebrovascular events [n (%)]  Acute kidney injury [n (%)]  Permanent pacemaker [n (%)]  New-onset LBBB [n (%)] | 82±5.1  108 (36.4)  27.3±4.8  204 (68.7)  69 (23.2)  248 (83.5)  100 (33.7)  177 (59.6)  172 (57.9)  122 (41.1)  1.4 (1.2-1.7)  56.7 (54.1-59.2) 53.3 (49.9-56.7)  0.7 (0.6-0.9)  12.0 (11.8-12.2)  3354 (1246-5462)  43.5 (29.1-57.9)  86 (29.0)  10 (3.4)  34 (11.4)  15 (5.1)  23 (7.7)  23 (7.7)  14 (4.7) | 81 ± 6.7  61 (69.3)  27.1 ± 4.6  62 (70.5)  21 (23.9)  68 (77.3)  33 (37.5)  55 (62.5)  54 (61.4)  39 (44.3)  1.4 (1.2-1.6)  56.5 (51.5-61.4)  55.6 (49.9-61.2)  1.3 (0.8-1.7)  12.8 (12.4-13.1)  7109 (3786-10.432)  79.1 (43.5-201.6)  28 (31.8)  5 (5.7)  13 (14.8)  2 (2.3)  10 (11.4)  7 (8.0)  7 (8.0) | 82± 5.8  89 (50.9)  27.4 ± 4.9  121 (69.1)  44 (25.1)  149 (85.1)  64 (36.6)  111 (63.4)  105 (60.0)  80 (45.7)  1.5 (1.4-1.7)  55.9 (51.8-60.0) 58.9 (53.9-63.9)  0.9 (0.8-1.2)  12.1 (11.8-12.3)  5669 (3265-8074)  53.7 (15-122.4)  128 (73.1)  12 (6.9)  37 (21.1)  8 (4.6)  22 (12.6)  11 (6.3)  10 (5.7) |  | 81± 6.0  33 (56.9)  26.1 ± 4.3  41 (70.7)  11 (19.0)  44 (75.9)  19 (32.8)  44 (75.9)  34 (58.6)  20 (34.5)  1.5 (1.2-1.8)  53.6 (47.9-59.1)  61.4 (54.5-68.2)  1.6 (0.5-2.6)  12.2 (11.8-12.6)  15769 (10050-31642)  39.2 (39.2-39.2)  37 (36.8)  5 (8.6)  11 (19.0)  1 (1.7)  3 (5.2)  6 (10.3)  5 (8.6) | 0.191  <0.001  0.337  0.984  0.815  0.217  0.852  0.135  0.935  0.455  0.884  0.836  0.120  0.010  0.004  0.003  0.692  <0.001  0.218  0.035  0.515  0.200  0.783  0.527 |

Table S1: Baseline clinical characteristics in subgroups: patients with preserved (>50%) and reduced (≤50%) LVEF and transfemoral (TF) or transapical (TA) approach. Total number and (%) or median with (interquartile range).

BMI, body mass index; eGFR, estimated glomerular filtration rate; LBBB, Left bundle branch block; NYHA, New York Heart Association.


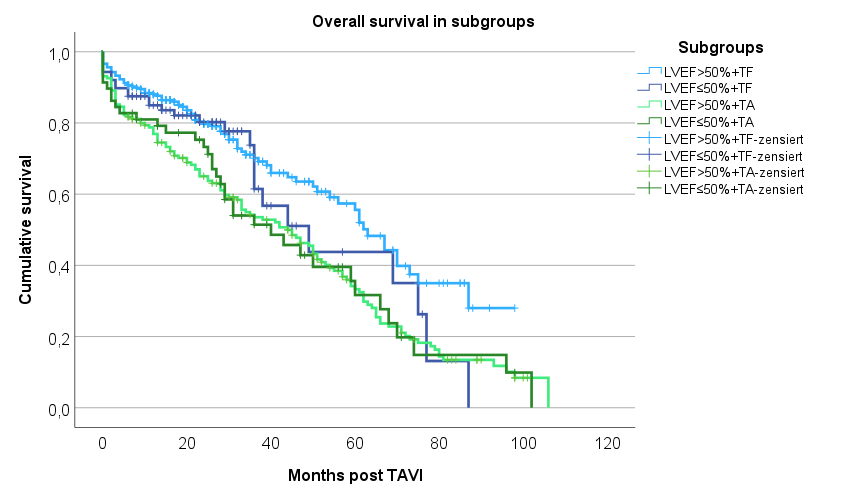


|  | | | | | | | | | |
| --- | --- | --- | --- | --- | --- | --- | --- | --- | --- |
|  |  | LVEF>50%+TF | | LVEF≤50%+TF | | LVEF>50%+TA | | LVEF≤50%+TA | |
|  |  | Chi-Quadrat | Sig. | Chi-Quadrat | Sig. | Chi-Quadrat | Sig. | Chi-Quadrat | Sig. |
| Log Rank (Mantel-Cox) | LVEF>50%+TF |  |  | 1,070 | ,301 | 18,153 | <,001 | 7,959 | ,005 |
|  | LVEF≤50%+TF | 1,070 | ,301 |  |  | 2,778 | ,096 | 1,462 | ,227 |
|  | LVEF>50%+TA | 18,153 | <,001 | 2,778 | ,096 |  |  | ,003 | ,958 |
|  | LVEF≤50%+TA | 7,959 | ,005 | 1,462 | ,227 | ,003 | ,958 |  |  |

Figure S1: Survival in subgroups: patients with preserved (>50%) and reduced (≤50%) LVEF and transfemoral (TF) or transapical (TA) approach. Kaplan-Meier curves and corresponding Log Rank tests.

|  | LVEF>50%+TF  N=297 | | LVEF≤50%+TF  N=88 | | LVEF>50%+TA  N=175 | | LVEF≤50%+TA  N=58 | |
| --- | --- | --- | --- | --- | --- | --- | --- | --- |
|  | HR (95% CI) | P value | HR (95% CI) | P value | HR (95% CI) | P value | HR (95% CI) | P value |
| Univariate Cox Regression  Cardiac Sizes  IVSd (mm)  LAA (cm2)  LAD (mm)  LVMi (g/m2)  LVPWd (mm)  RAA (cm2)  RVD_mid (mm)  Systolic function  MAPSE lateral (mm)  MAPSE septal (mm)  TAPSE (mm)  DD  Moderate to severe vs. mild  Lateral E/E‘  Septal E/E‘  sPAP (mmHg)  Multivariate Cox Regression  MAPSE septal (mm)  TAPSE (mm)  Septal E/E‘  sPAP (mmHg) | 0.971 (0.866-1. 089)  1.022 (0.989-1.055)  1.029 (0.993-1.066)  1.001 (0.994-1.008)  0.996 (0.894-1.110)  1.018 (0.982-1.055)  1.007 (0.969-1.047)  0.889 (0.811-0.976)  0.803 (0.717-0.900)  0.938 (0.897-0.982)  1.413 (0.913-2. 188)  1.017 (0.093-1.041)  1.029 (1.00 8-1.049)  1.026 (1.01 0-1.042)  0.852 (0.740-0.982)  0.972 (0.919-1.028)  1.017 (0.994-1.040)  1.021 (1.004-1.039) | 0.621  0.188  0.122  0.752  0.946  0.340  0.723  0.013  < 0.001  0.006  0.121  0.168  0.005  0.001  0.027  0.318  0.146  0.015 | 1.113 (0.908-1.334)  1.044 (0.982-1.110)  1.063 (0.999-1.131)  0.999 (0.986-1.012)  1.239 (0.962-1.597)  1.061 (0.999-1.127)  1.004 (0.947-1.064)  1.119 (0.928-1.348)  0.897 (0.671-1.198)  0.994 (0.925-1.067)  1.677 (0.627-4.481)  1.006 (0.972-1.040)  1.006 (0.979-1.034)  1.002 (0.976-1.028) | 0.248  0.171  0.054  0.858  0.097  0.056  0.893  0.239  0.462  0.858  0.303  0.747  0.667  0.891 | 1.026 (0.947-1.112)  1.024 (0.999-1.050)  1.021 (0.993-1.049)  1.000 (0.995-1. 005)  1.023 (0.930-1.125)  1.020 (0.997-1.043)  1.016 (0.985-1.048)  0.943 (0.868-1.025)  0.963 (0.882-1.052)  0.965 (0.924-1.007)  1.323 (0.907-1.930)  0.984 (0.961-1. 008)  1.004 (0.985-1. 023)  1.013 (1.00 0-1. 026) | 0.531  0.058  0.137  0.981  0.643  0.093  0.304  0.167  0.408  0.103  0.146  0.187  0.697  0.051 | 0.965 (0.802-1.159)  1.007 (0.958-1.058)  1.011 (0.956-1.060)  0.994 (0.984-1.003)  0.991 (0.814-1.208)  1.013 (0.955-1. 075)  0.989 (0.944-1.035)  0.973 (0.844-1.122)  0.858 (0.710-1.036)  0.896 (0.813-0.988)  0.952 (0.479-1.894)  0.9067 (0.924-1.012)  1.011 (0.977-1.046)  0.994 (0.967-1.022) | 0.701  0.789  0.644  0.178  0.931  0.658  0.631  0.706  0.111  0.027  0.889  0.147  0.546  0.657 |

Table S2: Cox Regression for echocardiographic predictors in subgroups: patients with preserved (>50%) and reduced (≤50%) LVEF and transfemoral (TF) or transapical (TA) approach. Hazard ratio (HR) with 95% confidence interval (CI).

DD: diastolic dysfunction; E/e’ ratio, the ratio of early diastolic filling velocity to mitral annular velocity; e´, early diastolic filling velocity; IVSd, interventricular septum wall thickness at end-diastole; LAA, area of left atrium; LAD, diameter of left atrium; LVMi, left ventricular mass index; LVPWd, left ventricular posterior wall thickness at end-diastole; MAPSE, mitral annular plane systolic excursion; RAA, right atrial area at end-systole; RVD, right ventricular diameter at end-diastole; sPAP, systolic pulmonary artery pressure; TAPSE, tricuspid annular plane systolic excursion


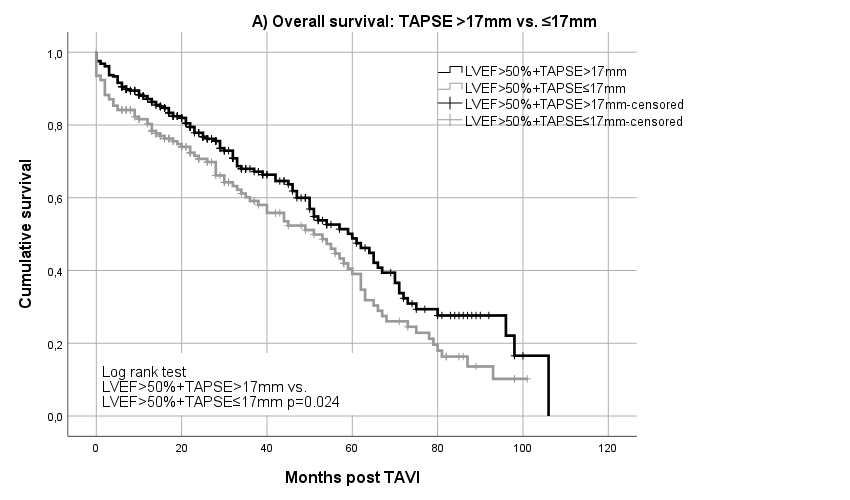

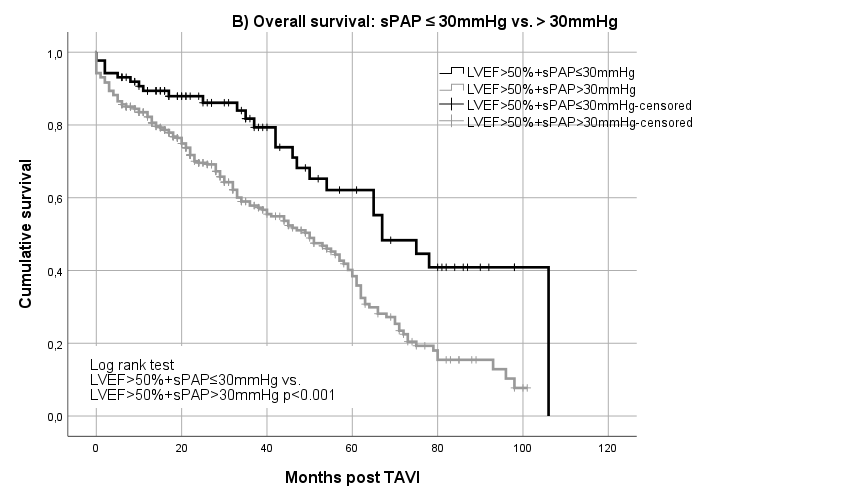


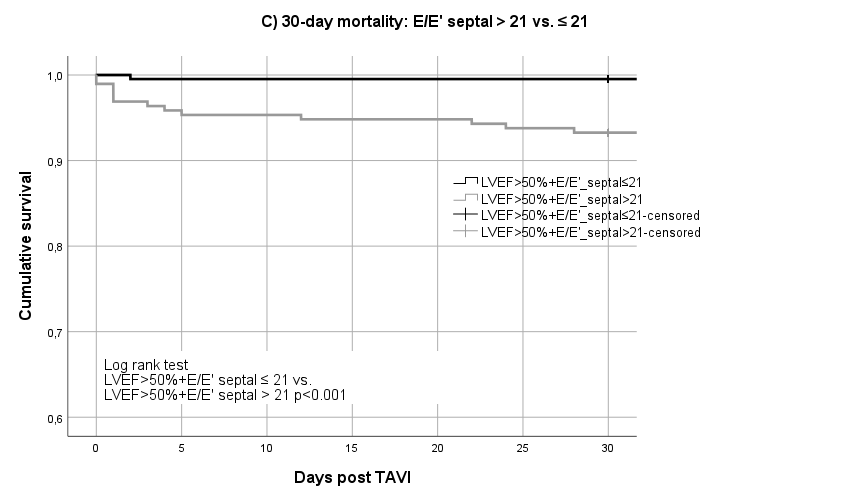

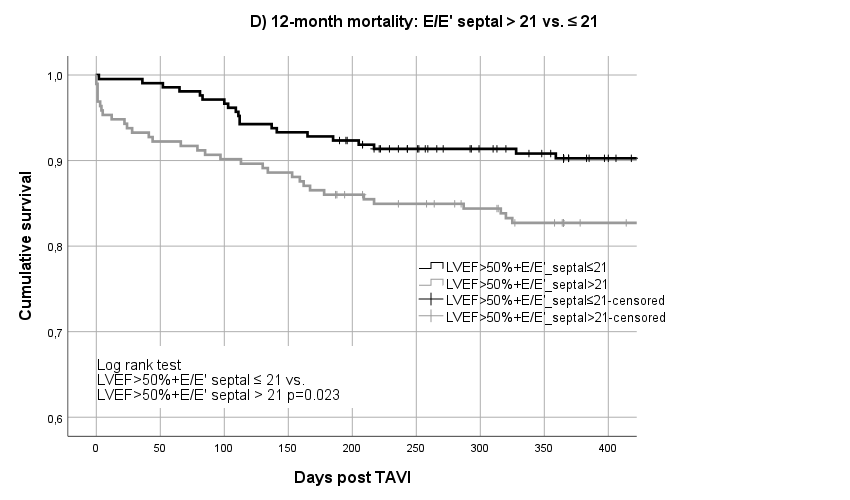


Figure S2: Survival curves for echocardiographic predictors in TAVI patients with preserved (>50%) LVEF. Overall survival: A+B, 30-day mortality: C, 12-month mortality: D


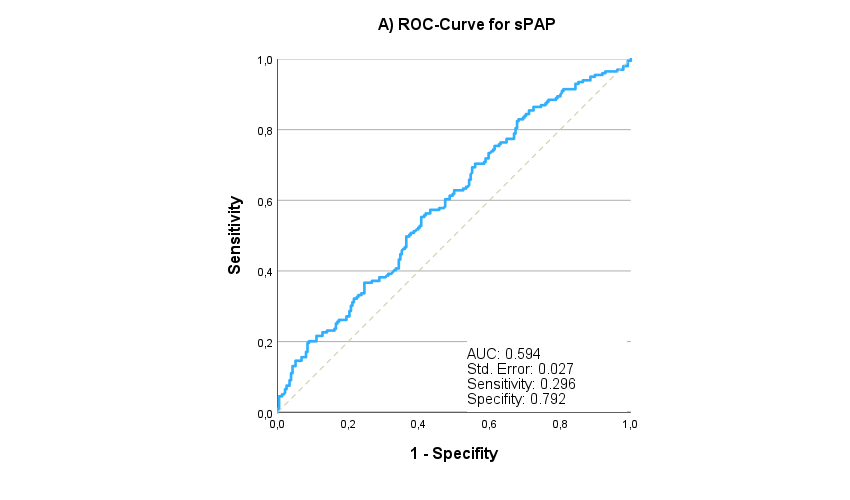

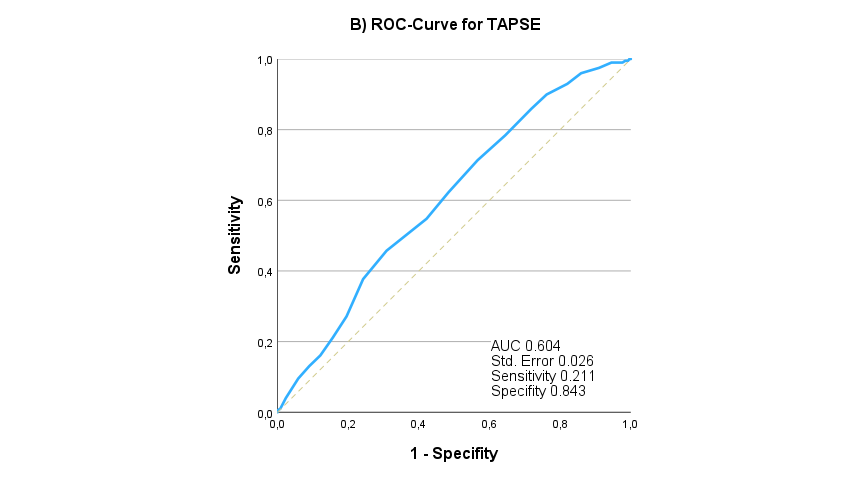


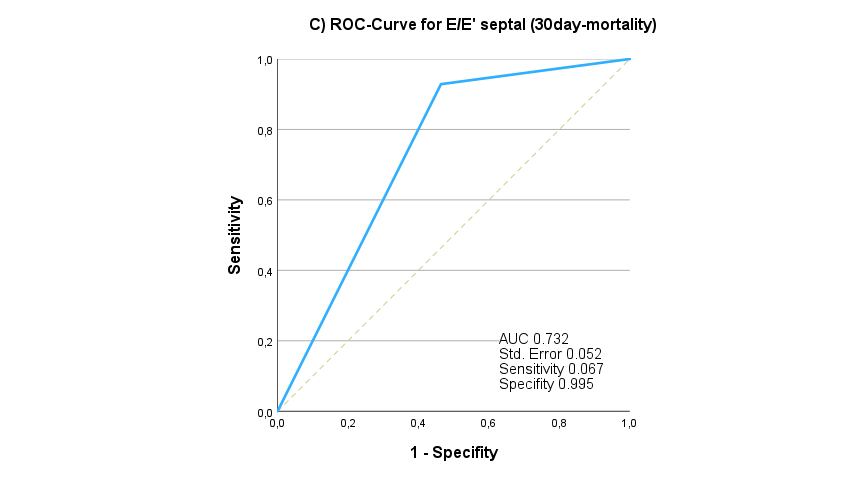

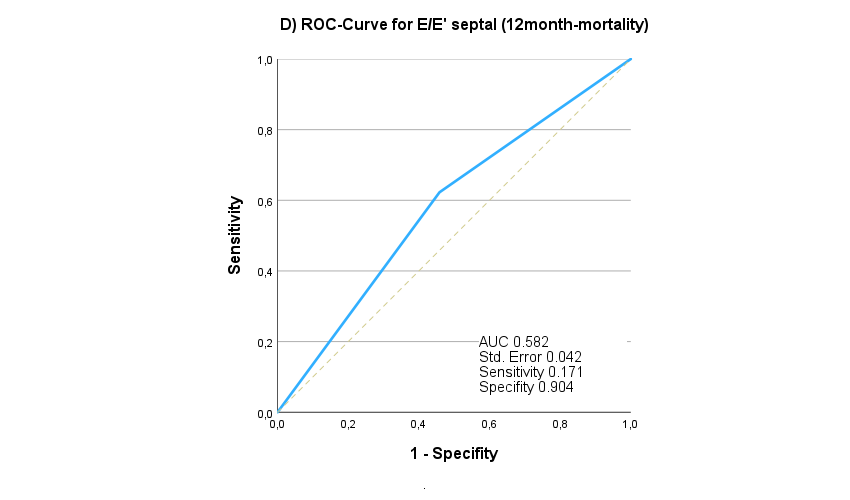


Figure S3: ROC Curves for echocardiographic predictors
